# Audio-visual speech-in-noise tests for evaluating speech reception thresholds: A scoping review

**DOI:** 10.1371/journal.pone.0338600

**Published:** 2026-01-27

**Authors:** Adeel Hussain, Adele M. Goman, Mandar Gogate, Kia Dashtipour, Jasper Kirton-Wingate, Zain Hussain, Aziz Sheikh, Michael A. Akeroyd, Amir Hussain

**Affiliations:** 1 Edinburgh Napier University, Edinburgh, United Kingdom; 2 School of Health and Wellbeing, University of Glasgow, Glasgow, Scotland, United Kingdom; 3 Nuffield Department of Primary Care Health Sciences Radcliffe Observatory Quarter, University of Oxford, Oxford, United Kingdom; 4 Hearing Sciences, School of Medicine, University of Nottingham, Nottingham, United Kingdom; Sri Ramachandra Institute of Higher Education and Research (Deemed to be University), INDIA

## Abstract

**Objective:**

To evaluate the advancements in speech intelligibility testing over the recent decades, with a particular emphasis on the development of audiovisual speech in noise tests that incorporate both auditory and visual modalities for the measurement of speech recognition thresholds.

**Design:**

A scoping review was conducted systematically to examine the existing literature on speech intelligibility testing methods. Following comprehensive screening process, studies were selected for detailed analysis, focusing on audiovisual integration and potential for remote or automated administration within studies methodologies.

**Study Sample:**

The review encompassed 11 scholarly articles that investigated diverse approaches to speech intelligibility testing.

**Results:**

The analysis revealed variability in the accuracy and reliability of speech intelligibility testing methods. Although certain methods demonstrated efficacy in incorporating audiovisual cues, none of the reviewed studies included provisions for remote administration, thereby necessitating the presence of a clinician for test execution. This limitation underscores the imperative for further research development of remote testing methodologies that leverage audiovisual technologies to assess speech in noise.

**Conclusions:**

The findings of this review underscore the critical need for advancement in speech intelligibility testing methodologies particularly integrating audiovisual components and enabling remote administration. The development in this domain holds significant potential to enhance the assessment and implementation of assistive technologies for individuals with hearing impairments.

## Introduction

Speech perception is a complex multimodal process that integrates both auditory and visual cues, exemplifying multimodal or multisensory integration, wherein various unisensory modalities such as sight, hearing, or touch are combined. Research has demonstrated that language processing is highly interactive, involving the combination of diverse information sources [1]. Speech, generated by the vocal apparatus, is filtered through the configuration of articulatory organs. There is an inherent and perceptible link between the auditory and visual properties of speech, since articulators such as lips, teeth, and the tongue visibly contribute to the process [2–4].

Extensive research in this domain has investigated the phenomenon by which listeners unconsciously engage in lip reading to enhance speech intelligibility in noisy environments [5–8]. Speech intelligibility assessments are generally conducted in clinical or research laboratory settings, where controlled conditions are maintained. However, this presents challenges when attempting to generalise these findings to real-world listening environments, which are characterised by variables such as ambient noise, environmental factors such as wind and machinery noise, and presence of multiple concurrent conversations [9].

In the context of audiology and hearing loss, it is currently estimated that around 5% of the global population, equivalent to 430 million people, experience hearing impairment [10]. The prevalence of hearing loss increases noticeably with over 25\% of individuals aged 60 and above affected by disabling hearing loss, which refers to hearing loss greater than 40 dB in the better-hearing ear in adults and greater than 30 dB in the better hearing ear in children [10,11]. Despite continuous advancements in research and technology, currently hearing assessments predominantly rely on audio-only (AO) methodologies.

Pure tone audiometry (PTA) is standard procedure for the identification and assessment of hearing loss and its severity. This diagnostic approach enables clinicians to accurately determine the extent of hearing impairment, thereby facilitating informed counseling and the provision of tailored recommendation to patients. PTA is widely regarded as the gold standard and most frequently employed test for detecting of hearing loss [12,13]. Although PTA provides essential data regarding a listener’s hearing sensitivity, Parmar et al. [14] found that hearing healthcare professionals view speech testing as particularly valuable for offering patients relatable insights into their functional hearing abilities. This information is crucial for guiding hearing aid fittings and constitutes as vital component of the comprehensive diagnostic test battery. These assessments are conducted under both aided and unaided conditions, encompassing evaluations in quiet as well as in the presence of background noise.

Speech-in-noise (SIN) tests are effective tools for assessing hearing loss across diverse populations and languages. A wide array of commercially available audio-only (AO) SIN tests are currently available, many of which are suitable for both adults and children. The AO SIN tests evaluate performance at the sentence, word, or phonemic level and include, both adaptive and fixed signal-to-noise ratio (SNR) tests are available. Fixed SNR tests include the Connected Speech Test (CST) [15] and the Speech Perception in Noise Test (SPIN) [16]. Adaptive tests, such as the Hearing In Noise Test (HINT) [17], Quick Speech In Noise (QSIN) [18], the Words-in-Noise Test (WIN) [19] and the Bamford–Kowal–Bench (BKB) SIN test [20], are commonly employed in clinical settings. These tests typically involve a target speaker delivering the specific material (sentence, word, or phoneme) amid background noise, which varies depending on the test. Background noises range from multi-talker babble to speech-shaped noise, with adaptive testing featuring variable noise or speech level, while fixed SNR tests maintaining constant noise levels. Stimuli may be presented through headphones to provide ear-specific results or via soundfield loudspeakers. Participants are instructed to repeat what they hear, and clinicians score the response based on the number of accurately recognised keywords, determining the percentage of correct words or the speech recognition threshold at 50% intelligibility, depending on the test employed.

Although the benefits of employing speech testing for guiding hearing aid fitting and as a component within a diagnostic test battery are well recognised, the widespread adoption of this practice remains limited. Certain countries [14], such as Canada and India, recommend speech testing as an essential component of audiology practice, whereas others, such as the UK, do not. Parmar et al. [14] have identified a lack of clinical time, inadequate training, and insufficient equipment as key factors contributing to the limited implementation of speech testing within a diagnostic battery, a trend particularly evident in the UK and likely to the observed global variability in service provision [14].

In addition to the limited adoption of SIN testing, current testing protocols exhibit several others limitations, including their failure to replicate real world scenarios [21] and the lack of integration of visual cues, which could enable listeners to benefit from auditory and visual information. The incorporation of visual cues into speech perception tests has been extensively investigated with a substantial body of research establishing that speech comprehension is significantly enhanced when both auditory and visual modalities are engaged [5,7,22,23]. This research consistently demonstrates the advantages of incorporating visual cues, particularly in environments where auditory signals are degraded. For instance, a study conducted by [7] with normal hearing adults revealed a marked improvement in word recognition when both auditory and visual cues were presented, compared to auditory input alone. In particular, the inclusion of visual speech information was found to enhance performance to a level equivalent to a 15 dB increase in SNR over AO conditions. Similarly, Gagné and Wittich [24] underscored the importance of visual cues for older adults with hearing loss, reporting an average 18% improvement in speech recognition when visual information was incorporated. These findings underscore the imperative to integrate visual cues into SIN testing protocols, particularly for populations where auditory processing alone may be insufficient, thus ensuring more accurate assessments of speech perception in realistic listening environments. Another critical consideration when integrating visual elements into speech tests is the specific contribution of visual input and the extent of its benefit. As noted by Tye-Murray et al. [25], lipreading (visual-only speech perception) plays a significant role in audiovisual (AV) speech perception, accounting for up to 60% of the variance in individual AV speech perception measures.

Expanding on the integration of visual cues in speech testing, it is also essential to consider the role of Speech Reception Threshold (SRT) and other speech perception measures in evaluating auditory performance. The SRT is a widely utilised metric in AO SIN tests that determines the minimum SNR at which a listener can correctly identify speech 50% of the time. Fixed SNR tests, which assess the percentage of correct responses at a predetermined SNR, offer the advantage of providing a straightforward evaluation of hearing aid benefit, facilitating patients comprehension. However, a critical limitation of these tests is the difficulty of selecting an appropriate SNR. If the SNR is set too low, the results may underestimate the true benefit of the hearing aids. Conversely, if the SNR is set too high, the perceived benefit may be overstated. This issue is particularly salient among high-performing cochlear implant users, where traditional fixed SNR tests may result in ceiling effects, thus failing to accurately differentiate level of performance. In contrast, adaptive SRT tests, which dynamically adjust the SNR based on the listener’s performance, offer more nuanced assessment capable of better distinguishing performance across a wide range of abilities [26]. This adaptive approach is essential for capturing the full spectrum of auditory processing capabilities and ensuring that the results are both meaningful and reflective of real-world listening conditions. While percentage-based tests and SRT measurements offer different perspectives on speech perception abilities, SRT testing can provide more fine-grained insights into specific aspects of hearing performance. Both approaches are interconnected, as an SRT can be derived from a psychometric function of percentage correct versus decibel level, and conversely, a percentage-versus-decibel curve can be calculated from an adaptive SRT test. The choice between these methods often depends on the specific experimental focus and goals. The implementation of adaptive SRT test in noise would serve as a valuable tool for assessing hearing capabilities and comparing hearing aid systems in both clinical and research settings, as these tests reveal variation in psychometric function slopes and offer a more comprehensive range of performance levels [26,27].

Traditionally, hearing services have predominantly been provided in hospitals or clinics, where testing is conducted by healthcare professionals. This centralised model of care presents notable limitations in accessibility, particularly for certain populations and specific circumstances. The shortage of qualified professionals, especially in low- and middle-income countries, constitutes a significant barrier to access [28]. Recent global insults, such as the COVID-19 pandemic, have underscored another critical limitation of clinic-based services: their susceptibility to disruption during public health crises. Safety measures, including lockdowns implemented to control the spread of infectious diseases, can severely impede access to conventional hearing healthcare services [29]. In response to these limitations and a need to enhance accessibility to hearing assessments, there has been a increase shift towards the development of remote and mobile-based AO SIN tests. These innovative approaches seek to democratise access to hearing screening and assessment tools, enabling individuals to undergo preliminary evaluations without necessitating in-person clinic visits. Several AO SIN tests have been adapted or newly developed with remote capabilities, using internet-based platforms or smartphone applications [30–32]. These tools hold significant potential for the widespread screening and monitoring of hearing health, particularly in underserved areas or during periods when physical access to healthcare facilities is restricted.

This comprehensive review aims to critically examine the evolution and current state of speech intelligibility testing, with a particular focus on SRT assessments that have integrated AV elements over the recent decades. The scoping review will systematically identify and evaluate studies that have incorporated visual components into SRT assessments, exploring their methodologies, outcomes, and potential clinical applications. Additionally, the review will investigate the extent to which these AV SRT tests have been adapted for remote administration, addressing a critical gap in accessibility for individuals in geographically isolated regions or those with technological or mobility constraints that hinder access to traditional clinical settings. Furthermore, this review will not only provide a comprehensive overview of the current landscape of AV SRT testing, but also to critically analyse the potential of these methods to enhance diagnostic accuracy, thereby informing development of an AV SIN test. Drawing on the findings of this scoping study, recommendation can be formulated regarding the applicability of existing methodologies or stimuli in development of a new remote British English AV SIN test. Consequently, this review underscores the necessity for future research to focus on the developing and validating AV speech tests that can be administered remotely or via mobile applications, while ensuring the reliability and validity of clinical assessments. Such advancements hold the potential to significantly improve to accessibility, comprehensiveness, and patient centreness of hearing healthcare services and could facilitate the future development of multimodal hearing aids by manufacturers.

## Methods

A scoping review was conducted, following the methodology outlined by Arksey and O’Malley [33]. These findings were systematically presented in the following sequence: (1) define a research question, (2) identify pertinent studies, (3) select relevant studies, (4) charting the data, and (5) collating, summarise, and reporting the results. This review adhered to the guidelines of the preferred reporting items for scoping reviews (PRISMA-ScR) [34].

### Identifying the research question

The primary research question addressed was:’Have any previously developed or researched AV SIN tests been used to measure SRT?’ A secondary question investigated which of these tests incorporated remote or automated functionalities?

### Identifying relevant studies

In this phase of study, we sought to establish the criteria for selecting publications to be included in the scoping review. Although scoping studies are inherently broad in scope, we deliberately identified specific criteria to guide our search process. The search strategy utilised for electronic databases was formulated based on our research questions and key concept underpinning the study. Prior to commencing the searches, two authors (AH and AG) reached a consensus on the relevant keywords for article retrieval. Searches were conducted from earliest records available up to February 18, 2025. The databases selected for this review included the Cochrane Library, IEEExplore, PubMed, Science Direct, and Web of Science, chosen for their comprehensive coverage of topics pertinent to health, engineering, social sciences, and psychology. Repositories containing grey literature were excluded from this systematic search, due to the absence of peer review which raised concerns regarding the authenticity, reliability, and reproducibility of the included work [35]. This exclusion was enforced to ensure that the retrieved literature would directly contribute to addressing the research question. The search terms employed in the database queries included “speech in noise,” “speech intelligibility,” “speech perception,” “speech recognition in noise,” “audiovisual,” “audio-visual,” and “auditory-visual.” These terms were consistently applied across all databases using boolean operators to structure the queries. Specifically, the key terms within each axis were combined using the “OR” operator and the search strategies for both axes were linked using the “AND” operator. The search commands were as follows: (“speech in noise” OR “speech intelligibility” OR “speech perception” OR “speech recognition in noise”) AND (“audiovisual” OR “audio-visual” OR “auditory-visual”). To minimise the risk of excluding relevant studies, no restrictions were placed on publication dates or languages.

### Study selection

All studies published in English that addressed AV SIN tests which used SRT as measurement were considered for inclusion in this review. The primary objective was to identify any AV SIN tests that had been developed without imposing any restrictions on the date range for inclusion. Additionally, no limitations were placed on sample size or study design, as the focus was on analysing the methodologies and development employed in previous research within the scope of this review.

Incomplete papers, opinion pieces, book chapters, editorials, and grey literature were excluded from further review. The management of all search results citations including the identification and merging of duplicates was conducted using Paperpile LLC 2024 software. Subsequently, three stage selection process was implemented to evaluate the articles.

The inclusion criteria were established based on the following parameters: (1) studies involving AV SIN testing (2) no age restriction for participants, (3) measurement of the SRT, 4) no limitation on the language of stimuli, and (5) articles written in English. Articles excluded were: (1) measurements without SRT, (2) were not written in English, and 3) consisted of clinical commentaries, editorials, interviews, letters, newspaper articles, abstracts only, or non-peer-reviewed literature (e.g., thesis).

During stage 1, two authors (AH and KD) independently reviewed the titles and abstracts of all articles related to AV SIN tests. In the second stage, the full-text articles were meticulously examined by the same authors (AH and KD) to assess their eligibility. The final stage involved a third author (AG) who evaluated the articles flagged by the previous reviewers, due to discrepancies, resolving any disagreement to make the final determination on inclusion.

**Fig 1:**
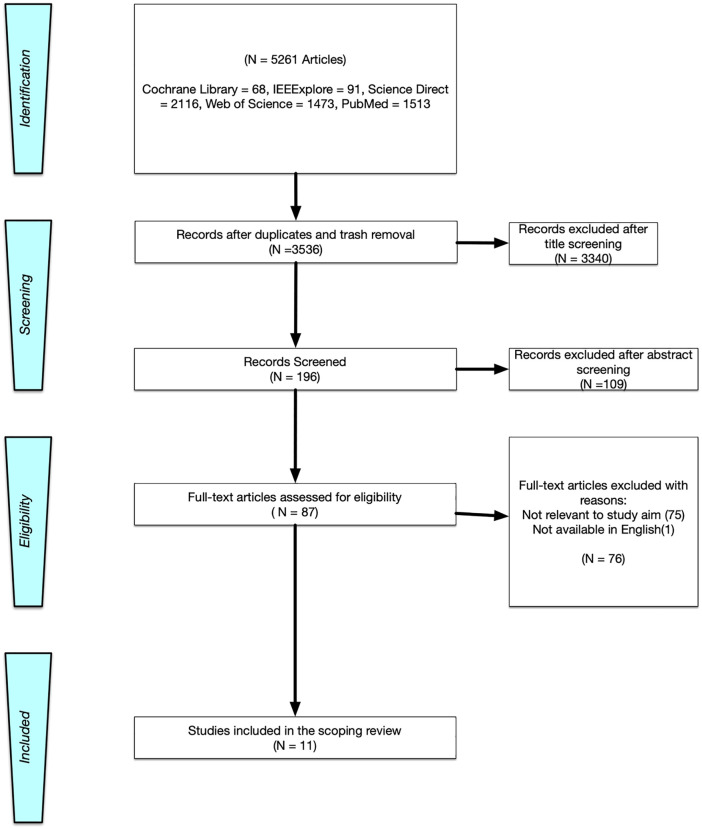
A PRISMA flow chart that shows the process of article selection [48].

Ultimately, fully eligible articles were chosen for further analysis and synthesis (Fig 1).

### Charting the data

The subsequent phase involved systematically organising the data extracted from the primary research reports under review. The data was structured according to the main research question to emphasise the primary findings. Each paper was meticulously read multiple times to ensure a comprehensive understanding of its aims, objectives, and findings, to ensure no relevant information was overlooked. The following information was systematically documented from each article: author(s), year of publication, study aim, research context, study participants, outcome measures, stimuli, language, test procedures and main findings.

### Collating, summarising and reporting results

Papers meeting the inclusion criteria were meticulously examined, with their content subjected to rigorous analysis and assessment. Recurring themes, the various methodologies employed for recording the AV material, and the procedures utilised for determining SRT were critically scrutinised and presented in the following section.

## Results

### Study selection

Articles were retrieved from five online databases, resulting in an initial set of 5261 papers. After eliminating duplicates and excluding irrelevant studies, 3536 articles remained. Title screening then led to the exclusion of 3340 articles, followed by abstract screening which further excluded 109 articles. This process resulted in a final selection of 87 articles for a comprehensive full-text review. Throughout this process, 76 articles were excluded based on the inclusion criteria due to reasons such as not having an SRT measurement, lack of AV integration or not being written in English. Consequently, the final number of studies included to address the research question was 11.

### Study characteristics

The following reviews each of the 11 included studies, summarising their aims, methodologies and findings (Table 1).

**Table 1:** Study characteristics and main findings.

Author, Year, Country	Aim	Remote	Participants	Measures	Stimuli	Procedure	Findings
Le Rhun et al, 2024 [42], France	To create and validate an audio-visual (AV) version of the French Matrix Sentence Test (FrMST) for evaluating speech intelligibility and lipreading ability.	No	35 young, normal-hearing participants (gender-balanced), aged 20–29 years (mean: 24.4 years), with no known hearing problems, normal or corrected vision, native French speakers.	speech reception thresholds (SRTs) of 80%	Target Stimuli: Female version of the French MST Noise Stimuli: long-term stationary noise of the average speech spectrum (LTASS)	Noise stimuli was kept constant at 65 dB SPL. Target stimuli was first presented at 25 dB SPL, adaptive procedure changed the speech level depending on subjects’ responses.	The research successfully developed and validated an audio-visual version of the French Matrix Sentence Test by dubbing existing audio recordings. Results demonstrated that the created AV material maintained effectiveness comparable to other language versions while providing significant benefits over audio-only testing. The investigation revealed adding visual cues to auditory stimuli yielded substantial improvements in speech recognition thresholds – specifically 4.6 dB SNR enhancement in noisy conditions and 9.25 dB SPL improvement in quiet environments. Notably, the study identified the importance of targeting SRT80 instead of traditional SRT50 to prevent ceiling effects from participants with strong lipreading abilities.
Choudhary et al, 2023 [43], USA	The principal objectives of this study was to assessthe combined effects of increasing lip contrast and scaling heads and to investigate the impact of display resolution. The overarching objective is to enhance human speech perception in challenging, noisy environments.	No	21 participants, 15 males, 7 females, all participants reported normal vision and no visual or vestibular disorders.	Speech Reception Thresholds (SRTs) of 50% confidence high/low measured in self-assessed performance -likert scale: from 1 (very hard) to 7 (very easy)	Target Stimuli: Triplet of digits spoken by female in American English, only monosyllabic digits used Noise Stimuli: 8-talker babble, four male and females.	Noise stimuli was kept constant at 60dBA, adaptive procedure- first triplet presented repeatedly until triplet entered correctly increasing in 4dB steps, decreased by 2dB and second triplet presented, laddering was continued in 2dB increments, higher if incorrect, lower if correct. A minimum of 25 presentations, SRT was calculated as the average signal-to-noise ratio of the last 10 triplets.	In this study, it was initially observed that display resolution significantly influenced participants’ speech perception in noise, with higher display resolutions showing a positive correlation with improved speech perception. Secondly, the visually enhanced method, which involved incorporating up-scaled facial representation and simulated lipstick for increased contrast, demonstrated its efficacy in enhancing participants’ speech perception. Lastly, the study revealed a preference among participants for head scaling over increasing facial contrast within the range of facial enhancements investigated. This study did measure SRT within the testing; however, it was not the main focus of the paper.
Llorach et al, 2022, Germany [39]	The objective was to develop and validate an AV adaptation of the German Matrix Sentence Test (MST), utilising the pre-existing AO speech material.	No	28 young normal hearing participants	speech reception thresholds (SRTs) of 80%	Target Stimuli: Female version of the German MST Noise Stimuli: continuous test-specific noise (TSN)	Noise stimuli was kept constant at 65 dB SPL, Target stimuli was first presented at 60 dB SPL, the adaptive used varied the speech level dependant of subjects responses.	This study aimed to validate an AV adaptation of the German MST using pre-existing AO material. The findings reveal that dubbed video recordings maintain the validity of the original audio material and provide benefits comparable to naturally synchronised AV recordings in assessing speech intelligibility. The need for higher SRTs was emphasised due to ceiling effects linked with subjects speechreading abilities. For young, normal-hearing participants, AV stimuli provided a significant SRT benefit of 5dB SNR in test-specific noise and 7dB SPL) in quiet compared to AO stimuli.
Van de Rijt et al, 2019, Netherlands [41]	The objectives of this study were to investigate whether the incorporation of lipreading, derived from visual speech cues, contributes to improved speech recognition compared to relying solely on auditory information in individuals with normal hearing. Additionally, the authors assessed whether the principle of inverse effectiveness is applicable to the AV enhancement of complex word recognition. The principle of inverse effectiveness states that the multisensory enhancement from combining two weak unisensory stimuli is larger than the enhancement from combining two strong unisensory stimuli.	No	18 native Dutch-speaking adults, mean age = 26 years. Range = 21, 40. All screened for normal hearing levels, normal or corrected-to-normal vision	Speech Reception Thresholds (SRTSs) of 50% for both AO and AV conditions	Target Stimuli: Dutch female speech therapist Noise Stimuli: Female stationary noise with the same longterm spectrum as the speech, randomly chosen silences fixed between 5ms and 2ms.	Noise stimuli were maintained at a constant level of 65 dB SPL. The target stimuli were manipulated across five levels: −21, −16, −13, −10, and −5 dB SNR. Testing was conducted at each of the five SNR levels, and by modelling the psychometric function, the SRT at 50% was subsequently derived.	In this study, the results revealed that AV speech exhibited an average improvement of 2.6 dB in speech recognition compared to AO speech. The most significant benefit was observed at moderate SNRs ranging from −13 to −20 dB, further supporting the inverse effectiveness hypothesis.
Devesse et al, 2018 [44], Belgium	The aims of this study were to create virtual human speakers that deliver precise and beneficial visual speech cues, to assess the quality and realism of the visual speech information provided by these virtual humans through experiments evaluating speech reading abilities and speech intelligibility. Lastly, the study sought to demonstrate the potential use of these virtual human speakers in future research and clinical evaluation tools as an alternative to recording real speakers.	No	Speech reading: 5 participants, 1 Male and 4 females, ages from 24–56 years old, minimum of 1 year of speech reading experience Speech intelligibility: 35 normal hearing participants, 13 male, 22 female, ages 18–30 years old, mean age 22.	Speech reading condition: Percentage correct Speech intelligibility: SNR thresholds at 50%	Target Stimuli: Flemish/Dutch monosyllabic (MS) Consonant-vocal-consonant (CVC) words, Disyllabic (DS) words, List sentence material, male and female speakers used for speechreading condition and female for speech intelligibility.	Noise stimuli: Speech weighted noise & multi-talker babble noise, Speech reading: three blocks of speech materials presented, virtual human and video randomised across participants. Speech Intelligibility: Female virtual human presented in a simulated restaurant, performed in sound field, 0 degrees for target stimuli, second speaker above subjects head for background noise, starting with 60 dB A noise level and 48 dB A speech level, adaptively adjusting speech level by 2dB after each sentence dependant on subject response. The SRT was calculated by averaging the SNR from the last 5 sentences, plus an 11th fictional sentence. Conditions were tested twice, order was randomised.	In this study, the authors developed and assessed two realistic virtual human speakers. In the speechreading condition, 57–67% of words and sentences were correctly recognised, scoring lower than when viewing a video of a real person. In the speech intelligibility condition, there was an average improvement in intelligibility ranging from 1.4 to 18 dB. Despite the slightly lower intelligibility with virtual humans, the study demonstrates promise for a more ecologically valid assessment of speech perception in the future.
Schreitüller et al, 2018,[45] Germany	In this study, the authors aimed to introduce and validate a novel approach to assess AV speech perception. This involved augmenting an established speech test with synthetic visual articulations generated by the Talking Head system MASSY. The objective was to determine the utility of this standardised method in clinical applications, specifically in evaluating individual proficiency in lipreading, AV gain, and integration. These measures are deemed crucial for the diagnosis and treatment of hearing disorders.	No	28 participants, 14 experienced cochlear implant users, mean age of 47.4 years, 10 females and 4 males, 14 normal hearing individuals, mean age of 46.3 years, 8 females and 6 males.	Speech intelligibility: SNR Threshold Measured at 5%, 50%, 80% and 95%.	Target Stimuli: German OlSa Matrix sentences, male speaker Noise Stimuli: quasi-stationary speech-shaped noise (“olnoise”)	8 lists of 20 sentences each, (2 AO and 2 visual only), (2 AO and 2 AV lists). SNR starting not specified, SNR estimated to produce 50\% correct target speech recognition. This aligned with their model-based adaptive approach rather than using a fixed starting SNR.	This study presents and validates an innovative approach to AV speech assessment, integrating synthetic visual articulations from the Talking Head system MASSY with sentences from the German Oldenburg Speech Test. The outcomes indicated elevated visual only speech recognition among cochlear implant users in contrast to controls, age-associated decreases in lipreading proficiency, noteworthy AV improvement over unimodal modalities, and effective amalgamation of auditory and visual speech cues. These findings are consistent with prior research utilising live or recorded speech. Thus, this study affirms the effectiveness of the standardised synthesised articulation approach in quantifying and characterising AV speech perception abilities pertinent to hearing research and clinical applications.
Arnold et al, 2010 [36], UK/France	The aim of this work was to develop a Paediatric Audiovisual Speech Test in Noise (PAVT), evaluate benefits of adding lip-reading to auditory signals for CI children, address the lack of valid lip-reading assessment tools for CI children and create a PAVT with high face validity and reliability.	No	12 Participants, Mean age at implantation for participants was 3.7 years and mean age of 12 years old at testing.	Time taken: 5mins SNR thresholds are calculated based on the average of the last 6 SNR values for both AO and AV at 50%.	Target Stimuli: One boy and one girl (11 years old), words from the McCormick and English as a Second Language (E2L) toy tests, both with and without the carrier phrase (‘Show me’) Noise Stimuli: Multi-talker babble	20 words presented per test, adaptive SNR-start with 20dB SNR, initially a 10dB step size is used, after first reversal step size is decreased to 5dB, after second reversal to 2.5dB, stays at 2.5 till test end. Participants faced the subject screen with loudspeakers placed 1 meter in front.	A novel PAVT for cochlear implant users evaluates the impact of lip-reading on auditory signal. Testing, adaptable for lip-reading, employs an adaptive signal-to-noise ratio. In pilot testing with 12 cochlear implant users, each session lasted about 5 minutes. Video recordings were of acceptable quality. Lip-reading yielded an average signal-to-noise ratio gain of 8.3 dB. PAVT 2.0, an enhanced version, emerged from initial feedback. Future plans include validation on a larger subject pool and deployment to various centres.
Bernstein & Grant, 2009, USA [37]	The aim of this study was to quantify the benefit of fluctuating masker (FMB) on speech intelligibility across various noise backgrounds, including stationary noise, interfering talker, and modulated noise, and to compare the outcomes between normal-hearing (NH) and hearing-impaired (HI) listeners. The investigation explored the influence of visual speech cues on FMB when presented alongside auditory stimuli for both NH and HI groups.	No	13 participants, 5 NH, 2 male and 3 females, age range: 30–58 years with a mean of 44.4 years, 8 HI, all male, age range: 49–80 years, with a mean of 66.0 years.	SRT at 50% was compared across different masker types (stationary, interfering talker, modulated noise) and presentation modalities (AO vs AV)	Target Stimuli: IEEE sentences, female talker, American English Noise Stimuli: 3 different masker conditions: 1) Steady state speech-shaped Gaussian noise 2) Interfering talker from a single male talker 3) A one-talker (Female) speech-modulated noise	6 sentences presented per run, 3 runs per condition. Adaptive method employed where NH participants started at -15dB and -6dB SNR for HI, if < 4 keywords were correct, SNR increased by 3 dB, if ≥ 4 keywords were correct, SNR decreased by 9–15 dB randomly, sentences were presented repeatedly, adjusting SNR each time, until the track showed ≥4 keywords correct. The procedure was carried out for AO and AV modalities across 3 noise types.	This study revealed a diminished fluctuating masker benefit for HI listeners compared to NH controls when their performance was assessed at matched speech intelligibility levels. However, much of this apparent deficit was mitigated when groups were evaluated at equivalent SNRs in stationary noise. Nevertheless, HI listeners exhibited 1–5 dB less fluctuating masker benefit, suggesting potential suprathreshold deficits that may influence dip listening capacities to some extent. The findings imply that both AO and AV fluctuating masker benefits are predominantly influenced by baseline speech understanding in steady noise backgrounds across diverse populations.
Macleod & Summerfield, 1990, UK [49]	The objectives of this study were to establish a standardised test for effectively assessing AO and AV speech perception in noisy environments. The paper also provides norms and guidelines for the practical application of the test in clinical or research settings.	No	22 subjects with normal hearing for preliminary screening. 24 subjects for refining the lists, age ranges from 15–32 years, mean age of 22 years. 20 subjects for evaluation of final lists, age ranges from 16 to 30 years, with a mean age of 22 years, normal hearing, Snellen acuity spatial-contrast sensitivity. 24 subjects, age range from 24 to 78 years, with a mean age of 56 years.	SRT at 50% measured for AO and AV conditions	Target Stimuli: 150 British English sentences Noise Stimuli: White noise	Noise level was fixed at 60 dBA. SNR for starting AO condition −20 dB. SNR for starting AV condition −28 dB. Adaptive procedure adopted were SNR was increased by 2 db until 50% correct SRT was estimated by averaging SNRs used in the last 10 sentences. 15 sentences per list	In this study, a key discovery was the creation of reliable and sensitive test materials suitable for both AO and AV conditions, facilitating the estimation of SRTs within a reasonable timeframe. The study also introduced an adaptive procedure that produces dependable results within 15 sentences, along with guidelines for application in clinical and research settings.
Cox et al,1989 [38], USA	The objective of this study was to create and validate an AV rendition of the Connected Speech Test (CST), enabling the assessment of speech intelligibility in more authentic, face-to-face listening scenarios. The authors pursued two specific goals within the study: 1) to examine whether existing CST passage pairs maintain consistent difficulty when incorporating video, and 2) to develop a new standardized version of the CST for AV administration if disparities were identified between the AO and AV conditions.	No	26 participants with normal hearing thresholds, all reported normal/corrected -to-normal vision, age range from 23–50 years old, average age of 31 years.	The primary outcome measure was the within-subject standard deviation of passage pair/set scores under AO versus AV test conditions. Lower standard deviations suggest more consistent difficulty across passages. Scoring accuracy for speech at supra-threshold levels with a pre-set SNR of −5 dB and −7 dB was quantified as a percentage of performance. These percentages were subsequently converted to rationalised arcsine units.	Target Stimuli: 48 CST passages, each passage consisting of 10 sentences and a familiar topic. Noise Stimuli: Multi-talker babble	The CST is a fixed signal-to-noise ratio (SNR) test, with babble noise set at either −5 dB or −7 dB SNR. The speech passages were consistently maintained at a 41 dB Leq level. Six to eight practice passages were administered before the presentation of 24 test passage pairs. Testing was conducted using insert headphones coupled to the test ear with a foam plug, and the non-test ear was plugged. Subjects were seated 1 meter away from a 33 cm colour video monitor in a soundproof room. Each passage contains 25 scoring words	This study introduced the AV version of the CSTv3 and assessed its comparability to existing AO versions by examining within-subject scoring variability across passages. Initial findings revealed slightly higher variability in the AV administration (8.2 rau) compared to AO (7.3 rau), suggesting some inconsistencies due to the addition of visual cues. However, by organizing passages into balanced difficulty sets, the AV variability reduced to 5.17 rau, closely aligning with the 5.05 rau observed in AO presentation. The similar within-subject variability supports the consistent difficulty levels of the new CSTv3 when incorporating both auditory and visual speech components. This ensures the retention of norms and facilitates meaningful comparisons with earlier AO research. The outcomes affirm the suitability of the new CSTv3 as a reliable measure of everyday speech intelligibility under more realistic, face-to-face listening conditions.
Macleod & Summerfield, 1987 [40], UK	The primary objective of this study was to quantify the magnitude of the AV benefit in comparison to auditory-alone conditions, examining how this advantage varies based on the lipreading proficiency of individual subjects.	No	20 native speakers of British English, age ranges from 20–37 years, mean age of 22 years, all had normal or corrected to normal vision and audiometrically normal hearing in tested ear.	SRT measured until all 3 keywords identified correctly in both conditions.	Target Stimuli: Bamford-Kowal-Bench (BKB) sentence lists, recorded with male speaker with southern British accent Noise Stimuli: Broad-band Noise	Noise level was fixed at 60 dBA.SNR for starting audio condition was presented at -18dB. SNR for starting AO condition was presented at −30 dB. SNR was increased in 2 dB steps until the subject repeated sentence with 3 keywords correctly.	The key findings of the study indicate a substantial advantage in AO speech perception over AO conditions. This AO benefit was found to be correlated with individuals’ lipreading abilities. The results substantiate a straightforward model in which the AO advantage arises from heightened visual processing, specifically, while auditory and linguistic processing remain consistent across conditions.

### Stimuli and measures

An analysis of the studies revealed substantial variability in both the speech stimuli and measurement methodologies employed for AV SIN testing. The speech materials ranged from basic digit triplets to more intricate sentence-based stimuli. Several studies utilised standardised speech tests, such as matrix sentence tests, BKB sentences, and IEEE sentences, while others developed bespoke materials, including passages from the CST or novel sentence lists. In terms of measurement, most studies focused on determining SRTs, albeit with varying target percentages. Whilst many studies employed the conventional 50% correct performance criterion, others explored alternative thresholds. such as 80% SRT, whilst another study examined multiple thresholds, including 5%, 50%, 80%, and 95%. Furthermore, another study calculated SRTs based on the mean of the final six SNR ratio values. These variations in SRT percentages reflect ongoing efforts to optimise sensitivity and mitigate ceiling effects in AV testing. Adaptive procedures were commonly used for efficient SRT estimation, although some studies incorporated fixed SNRs to characterise performance across diverse listening conditions. Scoring methodologies encompassed both keyword and whole-sentence approaches (Table 2).

**Table 2:** Critical appraisal of methodological strengths and limitations of included studies.

Author, Year	Methodological Strengths	Methodological Limitations
Le Rhun et al, 2024 [29]	• Validated an AV version of a standardised, high-repetition test (French Matrix Sentence Test). • Addressed potential ceiling effects in AV conditions by targeting a higher, more sensitive threshold (80% SRT).	• Used dubbed video recordings over pre-existing audio, which can introduce synchronisation inconsistencies. • The sample consisted only of young, normal-hearing participants, limiting the generalisability of the findings to clinical or older populations.
Choudhary et al, 2023 [2]	• Employed an innovative design using a virtual speaker to systematically control and test visual enhancements like lip contrast and head scaling. • Investigated the impact of technical factors like display resolution, which is relevant for potential remote testing.	• Used digit triplets as stimuli, which offer poor ecological validity for representing real-world communication. • SRT was a secondary measure rather than the primary focus, and the confidence-based measurement is unconventional.
Llorach et al, 2022 [39]	• Successfully adapted a standardised test (German Matrix Sentence Test) for AV use, ensuring high comparability. • Also identified and addressed ceiling effects by recommending 80% SRT for AV testing, contributing to best practice.	• Relied on dubbed video, which may not be as natural as simultaneously recorded AV material. • The study was limited to young participants with normal hearing, restricting clinical applicability.
Van de Rijt et al, 2019 [41]	• Rigorously tested the principle of inverse effectiveness by measuring performance across a fixed range of SNRs, providing deep insight into the psychometric function of AV speech perception.	• The sample size was relatively small (n = 18). • Findings are limited to normal-hearing adults and may not apply to individuals with hearing impairment.
Devesse et al, 2018 [44], Belgium	• Pioneered the use of realistic virtual humans in a clinical context, offering excellent stimulus control and potential for creating ecologically valid test environments (e.g., a simulated restaurant)	• The virtual humans were found to be less intelligible than real human speakers, suggesting the technology requires further refinement. • The SRT calculation method was complex and atypical.
Schreitüller et al, 2018[45]	• Directly compared cochlear implant users with normal-hearing controls, providing high clinical relevance. • Integrated a standardised speech test (German OlSa) with a validated “Talking Head” system, ensuring a high degree of procedural control.	• The synthetic nature of the “Talking Head” may not fully capture the nuances of natural human visual speech cues.
Arnold et al, 2010 [36]	• Addressed a significant gap by developing an AV test specifically for a paediatric population, using age-appropriate stimuli. • The procedure was quick to administer (approx. 5 minutes), which is crucial for paediatric testing.	• Presented as a pilot study with a very small sample size (n = 12), requiring further validation on a larger cohort. • The paper noted that an enhanced version (PAVT 2.0) was needed, indicating limitations in the initial version.
Bernstein & Grant, 2009 [37]	• Included both normal-hearing and hearing-impaired listeners, strengthening the study’s clinical validity and generalisability. • Systematically compared different types of ecologically relevant maskers (e.g., interfering talker, modulated noise), which is critical for understanding real-world performance.	• Used IEEE sentences, which are phonetically balanced but offer a limited number of unique sentences compared to matrix tests, posing a challenge for repeated measures.
Macleod & Summerfield, 1990 [49]	• A foundational study that developed and standardised a reliable procedure for measuring AV SRTs. • Provided norms and clear guidelines for clinical and research application, establishing an early benchmark in the field.	• The stimuli were created with older technology, and the use of white noise as a masker has low ecological validity compared to modern speech-shaped or babble noise.
Cox et al,1989 [38]	• Utilised passages of conversational speech (CST), which offers very high ecological validity by mimicking real-world listening situations. • Validated the AV version against the established audio-only CST, ensuring continuity.	• As a fixed-SNR test, it is susceptible to floor and ceiling effects, especially among high-performing individuals. • Scoring conversational speech is inherently more complex and time-consuming than scoring keyword-based sentences.
Macleod & Summerfield, 1987 [40]	• Seminal early research that successfully quantified the benefit of visual cues and correlated it with individual lipreading ability, providing a theoretical model for the AV advantage.	• Used BKB sentences, which are simple and may not fully reflect the complexity of adult conversational discourse. • The use of broad-band noise as a masker is not representative of typical real-world listening environments.

### Visual integration

The studies reviewed utilised a range of methods to present visual speech information and assess its integration with auditory cues. Several studies employed video recordings of real speakers L. Arnold et al. [36]; Bernstein and Grant [37]; Cox et al. [38]; Llorach et al. [39, 40]; Van de Rijt et al. [41]; Le Rhun et al. [42] providing naturalistic visual speech cues that facilitated the integration process. In contrast, other studies employed virtual human speakers Choudhary et al. [43]; Devesse et al. [44]; Schreitmüller et al. [45], which despite sacrificing some realism, allowed for enhanced control over visuals elements such as lip contrast and head scaling. Although the extent of visual benefit varied across studies, it was consistently significant, with improvements in SRTs ranging from 1.5 to 5 dB when visual cues were incorporated. Furthermore, Van de Rijt et al. [41] and Bernstein and Grant [37] provided empirical support for the principle of inverse effectiveness, demonstrating that the benefit of visual cues was maximised at intermediate SNRs where AO performance was neither excessively high nor low.

### Remote testing

A key finding of this review was the lack of remote testing capabilities in the AV SIN tests examined. None of the 11 studies incorporated methods for administering tests remotely or via telehealth platforms. All tests were conducted in controlled laboratory or clinical environments, with participants required to be physically present for the testing sessions.

Future innovations could leverage virtual reality (VR) to create immersive and standardised testing environments or employ AI-generated, photorealistic avatars to offer precise control over visual speech cues, overcoming the inconsistencies of video recordings.

## Discussion

This comprehensive scoping review focuses on AV SIN tests with SRT measurements, aimed to (i) identify developed AV SIN tests specifically designed to measure SRT, (ii) evaluate the remote testing capabilities of these assessments. In analysing the search results, we identified methodologies employed in the development of various tests. Our investigation revealed 11 studies demonstrating considerable variability in functionality, with several requiring further development or validation. This highlights both significant progress in the field and areas necessitating future development. Due to our stringent screening criteria, the number of research studies included in this chapter is substantially lower than in comparable reviews [46] exploring AV speech perception. However, this focused sample enables us to thoroughly examine the methodologies utilised in previous studies and gain valuable insights into techniques that can be adapted and implemented in future research.

### Speech material and masking noise

The reviewed studies demonstrate significant progress in developing AV SIN tests across different languages and populations, using a diverse array of speech materials and masking noise. The studies included matrix sentence tests [39,42,45], which are beneficial due to their highly controlled vocabulary and syntactic structure, ease of adaptability across languages, and extensive number of possible combinations, rendering them suitable for repeated measures. Additionally, more naturalistic sentence materials [44] have been validated and standardised for clinical application, while word-level stimuli (L. [36]) represent an alternative approach.

Choudhary et al. [43] used digit triplets, which offer simplicity and rapid administration, thereby minimizing linguistic confounds. However, this material does not accurately reflect the complexities of real-world communication challenges, nor does it assess sentence-level processing. MacLeod and Summerfield [40] utilised BKB sentences, which are standardised and widely used in research. Despite their utility, these sentences may be constrained by the limited number of available material and may not adequately reflect the complexity of adult conversational discourse. Bernstein and Grant (2009) utilised IEEE sentences, which are phonetically balanced and commonly employed in speech testing. Although this corpus is known for its low context predictability, it could be limited by the number of sentences available when compared to matrix sentences (720 sentences versus the possibility of 100,000 possible sentence combinations). Cox et al. [38] used material from the CST test, which consists of 48 passages of conversational speech. While this test has high ecological validity, the use of conversational speech makes it more difficult to control for linguistic factors, and scoring can be more complex and time-consuming.

This variety reflects a tension between the need for controlled, comparable stimuli and the desire for ecological validity. Future development of AV SIN tests should strategically balance competing factors by incorporating a diverse range of speech materials. These materials should include single words and sentences, thereby enabling a comprehensive assessment of AV speech perception across multiple levels of linguistic complexity, from phoneme recognition to contextual comprehension.

The diversity of masking noises used across studies, including speech-shaped noise, multi-talker babble, and modulated noise, whilst some reflect the complexity of real-world listening environments others are used for laboratory experiments. While speech-shaped noise offers consistent energetic masking, it may not fully capture the informational masking effects encountered in everyday listening situations. The comparative analysis of various noise types within individual studies, as done by Bernstein and Grant [37], is particularly valuable in elucidating how different masker characteristics interact with AV integration processes. These maskers often fall into two categories; energetic masking, where noise overlaps spectrally with the speech signal, obstructing it at the auditory periphery and informational masking which arises from cognitive interference, such as competing speech signals that are perceptually similar to the target. Future AV SIN tests could benefit from incorporating adaptive technologies that manipulate both the type and intensity of background noise to offer a more comprehensive assessment of AV speech perception in challenging listening conditions.

It is critical to note that the studies identified in this review were exclusively conducted in Western countries, utilising materials in English, German, French, and Dutch. This reveals a significant geographic and linguistic gap in the literature. There is a notable absence of AV-SIN test development and validation in languages from Asia, Africa, and South America. Given that linguistic and cultural factors can significantly influence speech perception, the direct application of existing tests to diverse global populations is not appropriate. Future research should prioritise the development of culturally and linguistically adapted AV-SIN tests to ensure that the benefits of this assessment methodology are accessible globally and relevant to diverse patient populations.

### Procedural aspects and scoring methods

The review underscored several important methodological considerations for AV SIN testing, including the choice between adaptive procedures and fixed SNR measurements, as well as differences in scoring methods and the number of trials. Adaptive procedures for estimating SRTs offer efficiency and precision but may not capture the full range of performance across different SNRs. The approach taken by Van de Rijt et al. [41] of measuring performance across a fixed range of SNRs provides valuable insights into the shape of the psychometric function for AV speech perception, revealing important effects such as inverse effectiveness [41]. Inverse effectiveness is the principle that the benefit of combining auditory and visual information grows as the individual signals become less reliable.

Another crucial aspect to consider is the selection of an appropriate SRT percentage. Studies have demonstrated that a 50% threshold may be too easy to reach when visual cues are present due to the benefits of lipreading [39]. Scoring methods also varied, ranging from keyword scoring for sentences to more detailed phoneme-level scoring, each offering different balances between efficiency and the depth of information obtained. Additionally, studies differed in trial count, with test lists ranging from 10 to 30 sentences, underscoring the need for further research to find the optimal balance between test reliability and administration time.

Future advancement in AV SIN tests could benefit from integrating diverse approaches. Employing adaptive procedures to rapidly estimate SRTs while alongside fixed SNRs to characterise the full psychometric function, may enhance test accuracy. Additionally, incorporating multiple scoring levels could provide a comprehensive assessment, offering both overall intelligibility measures and detailed insights into AV integration.

### Visual integration

The exploration of both video recordings and virtual human speakers for presenting visual speech information signifies an important area of innovation in AV SIN testing. Although video recordings of real speakers currently offer the most naturalistic visual cues, the potential benefits of virtual humans, in terms of stimulus control and flexibility, make this a promising avenue for future research. An important consideration is the simultaneous recording of both visuals and auditory, as studies have shown that dubbing during post-processing can introduce inconsistencies [39, 42].

The consistent finding of significant visual benefits across studies, with SRT improvements ranging from 1.5 to 5 dB, underscores the importance of incorporating visual cues in speech intelligibility assessments. The evidence for inverse effectiveness observed in several studies has significant implications for test design and the results interpretation. Future AV SIN tests should be designed to capture this phenomenon, by adjusting SNRs to optimise AV integration for each individual. Additionally, further research is needed to examine individual differences in the capacity to integrate auditory and visual speech cues, which have meaningful implications for developing rehabilitative strategies in clinical populations.

### Remote testing

The absence of remote testing capabilities in current AV SIN assessments highlights a significant gap in the field. As technology has advanced, particularly in recent years, the potential for remote administration of speech perception tests has grown significantly. With the growing importance of telehealth in audiology and the global shift towards remote healthcare delivery [47,50], it is imperative to prioritise the development of AV SIN tests that can be reliably administered in remote settings. Leveraging improved connectivity and emerging telehealth platforms, future research may increasingly incorporate remote testing methods to address this need.

However, the transition to remote AV SIN testing presents several challenges. Ensuring consistent audiovisual presentation across diverse devices and varying internet connections is critical. Additionally, safeguarding test integrity in unsupervised settings poses unique difficulties. Potential solutions may involve the development of specialised software or web-based platforms for AV SIN test delivery, integrating automated calibration procedures to standardise device output, and employing advanced encryption and authentication technologies to protect test materials.

Rigorous research will also be necessary to validate remote versions of AV SIN tests by comparing them with in-person administration to ensure equivalence of results. The successful advancement of remote AV SIN testing capabilities would substantially enhance the accessibility and clinical applicability of these assessments, facilitating broader adoption in both research and clinical practice. This development has the potential to revolutionise speech perception testing, bridging gaps in accessibility and aligning with the broader trends in telehealth innovation.

### Strengths and limitations

A primary strength of this scoping review is its specific focus on SRTs measured through AV tests. This focus, however, inherently excluded a substantial body of research that examines AV speech perception without formally quantifying thresholds. Much of the research within the fields of psychology and neuroscience explores behavioural and neurological mechanisms underlying the integration of auditory and visual inputs during language processing without calculating speech reception scores. As a result, our review may have overlooked significant insights that this broader literature on AV speech perception could have provided. Nevertheless, we chose to focus specifically on SRTs because they offer a quantifiable and clinically relevant measure of speech intelligibility, which is particularly important for evaluating and comparing the performance of AV-based hearing assessments and interventions. This strategic focus allowed for the identification of AV assessments that were expressly designed and validated to measure speech intelligibility thresholds. Although the application of the SRT criterion resulted in a more limited pool of eligible studies, it allowed for a more focused mapping of AV testing methodologies specifically designed to quantify visual speech intelligibility gains. By narrowing the scope in this way, the review highlights the range of existing tools, their key characteristics, and areas where further methodological development or validation may be needed.

Furthermore, the decision to include only studies published in peer-reviewed journals may have introduced a degree of publication bias. Research on AV speech testing that is documented in unpublished manuscripts, conference proceedings, and dissertations was likely excluded. Expanding the scope of future reviews to include this so-called grey literature could yield additional perspectives and insights. An additional advantage of our inclusion criteria was the lack of restrictions regarding publication dates. This inclusive approach provided a comprehensive view of the development trajectory of AV speech testing, encompassing efforts that span several decades. By reviewing foundational works, we were able to identify early pioneers who explored the use of visual cues to enhance speech perception, striving towards the optimisation and validation of AV speech tests.

Similarly, our review identified a lack of research focusing on diverse populations. Although one study developed a test for paediatric use, most of the research cantered on monolingual adults with normal hearing or post-lingual hearing loss. The unique challenges faced by multilingual individuals, pre-lingually deaf children, or individuals with comorbid cognitive or visual impairments were largely unaddressed. The applicability of these findings to populations in low-resource settings, where access to technology and clinical expertise is limited, also remains unexplored. This limits the global generalisability of the current body of evidence and underscores the need for research that is more inclusive of diverse participant groups.

## Conclusions

In conclusion, this review identified 11 studies conducted in English, Dutch, French and German languages and specialised research domains, providing a comprehensive evaluation of existing AV SIN tests and methodologies. The analysis revealed substantial variability across studies highlighting the necessity for further research to achieve adoption of these tests. A key finding emphasised the importance of carefully calibrating scoring percentages and threshold criteria in future test designs to mitigate ceiling and floor effects, which may otherwise limit test sensitivity.

Significantly, none of the current AV assessments reviewed incorporate remote administration capabilities, underscoring the significant gap in the field. This presents a substantial opportunity to utilise telehealth innovations, thereby increasing accessibility to testing for populations constrained by geographic location, mobility issues, or availability. Additionally, careful considerations of the speech materials used in these assessments is critical to optimising reliability and validity. Factors such as the selection of talkers and linguistic complexity play a pivotal role in shaping test outcomes. Additionally, the design of adaptive procedures must carefully balance precision in threshold measurement, test efficiency, and the minimisation of participants fatigue or demotivation.

Therefore, the development a novel AV British English SIN test should prioritise addressing these identified gaps and consideration. The successful development of a remote test of this nature has the potential to transform clinical practice by facilitating more frequent assessments without the need for clinician involvement or specialised testing environments like soundproof booths. Moreover, such advancements, particularly those integrating telehealth platforms with emerging technologies like virtual reality and artificial intelligence, would be invaluable for researchers focused on developing ecologically valid assessments that address the limitations of current AO protocols and pave the way for truly patient-centred hearing healthcare.
